# Benzyl 3-[(*E*,*E*)-3-phenyl­prop-2-enyl­idene]dithio­carbazate

**DOI:** 10.1107/S1600536808013354

**Published:** 2008-05-10

**Authors:** M. T. H. Tarafder, K. A. Crouse, M. Toihidul Islam, Suchada Chantrapromma, Hoong-Kun Fun

**Affiliations:** a Department of Chemistry, Rajshahi University, Rajshahi 6205, Bangladesh; b Department of Chemistry, Universiti Putra Malaysia, 43400 Serdang, Selangor, Malaysia; c Department of Chemistry, Rajshahi University of Engineering and Technology, Rajshahi 6205, Bangladesh; d Department of Chemistry, Faculty of Science, Prince of Songkla University, Hat-Yai, Songkhla 90112, Thailand; e X-ray Crystallography Unit, School of Physics, Universiti Sains Malaysia, 11800 USM, Penang, Malaysia

## Abstract

The title compound, C_17_H_16_N_2_S_2_, a dithio­carbazate derivative, adopts an *EE* configuration with respect to the C=C and C=N double bonds of the propenyl­idine group. The 3-phenyl­prop-2-enyl­idene and dithio­carbazate fragments lie essentially in the same plane, with a maximum deviation from that plane of 0.074 (2) Å, while the dihedral angle between the 3-phenyl­prop-2-enyl­idene and the benzyl group is 77.78 (7)°. In the crystal structure, mol­ecules are linked by an N—H⋯S hydrogen bond and a weak C—H⋯S inter­action involving the terminal thione S atom, forming dimers that are arranged into sheets parallel to the *bc* plane. The crystal structure is also stabilized by C—H⋯π inter­actions.

## Related literature

For information on values of bond lengths, see Allen *et al.* (1987 ▶). For related structures of dithio­carbazate derivatives, see, for example: Crouse *et al.* (2004 ▶); Fun *et al.* (2008 ▶); Shanmuga Sundara Raj *et al.* (2000 ▶). For applications and bioactivities of dithio­carbazate derivatives, see, for example: Ali & Tarafder (1977 ▶); Ali *et al.* (2001 ▶, 2002 ▶, 2008 ▶); Chan *et al.* (2008 ▶); Chew *et al.* (2004 ▶); Crouse *et al.* (2004 ▶); Tarafder *et al.* (1978 ▶, 1981 ▶, 2001 ▶, 2008 ▶).
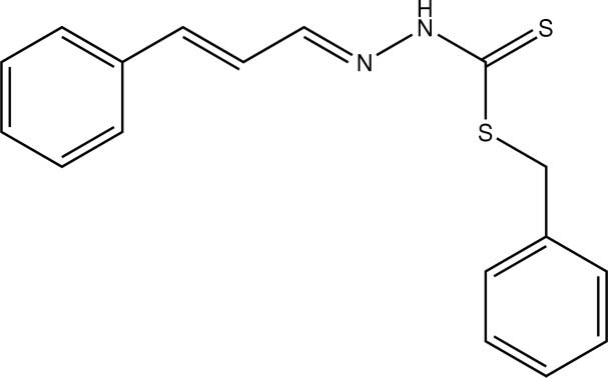


## Experimental

### 

#### Crystal data

C_17_H_16_N_2_S_2_
*M* *_r_* = 312.44
Triclinic, 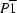

*a* = 5.4350 (3) Å
*b* = 11.6333 (7) Å
*c* = 13.6289 (8) Å
α = 66.869 (4)°
β = 82.723 (4)°
γ = 87.520 (4)°
*V* = 786.04 (8) Å^3^
*Z* = 2
Mo *K*α radiation
μ = 0.33 mm^−1^
*T* = 100.0 (1) K
0.58 × 0.19 × 0.05 mm

#### Data collection

Bruker SMART APEX2 CCD area-detector diffractometer
Absorption correction: multi-scan (*SADABS*; Bruker, 2005 ▶) *T*_min_ = 0.829, *T*_max_ = 0.982
16100 measured reflections
3570 independent reflections
2870 reflections with *I* > 2σ(*I*)
*R*_int_ = 0.044

#### Refinement

*R*[*F* ^2^ > 2σ(*F* ^2^)] = 0.035
*wR*(*F* ^2^) = 0.092
*S* = 1.07
3570 reflections
194 parameters
H atoms treated by a mixture of independent and constrained refinement
Δρ_max_ = 0.28 e Å^−3^
Δρ_min_ = −0.27 e Å^−3^

### 

Data collection: *APEX2* (Bruker, 2005 ▶); cell refinement: *APEX2*; data reduction: *SAINT* (Bruker, 2005 ▶); program(s) used to solve structure: *SHELXTL* (Sheldrick, 2008 ▶); program(s) used to refine structure: *SHELXTL*; molecular graphics: *SHELXTL*; software used to prepare material for publication: *SHELXTL* and *PLATON* (Spek, 2003 ▶).

## Supplementary Material

Crystal structure: contains datablocks global, I. DOI: 10.1107/S1600536808013354/sj2494sup1.cif

Structure factors: contains datablocks I. DOI: 10.1107/S1600536808013354/sj2494Isup2.hkl

Additional supplementary materials:  crystallographic information; 3D view; checkCIF report

## Figures and Tables

**Table 1:** Hydrogen-bond geometry (Å, °)

*D*—H⋯*A*	*D*—H	H⋯*A*	*D*⋯*A*	*D*—H⋯*A*
N1—H1*N*1⋯S2^i^	0.87 (2)	2.53 (2)	3.3714 (19)	165 (2)
C9—H9*A*⋯S2^i^	0.93	2.93	3.7264 (18)	144
C15—H15*A*⋯*Cg*1^ii^	0.93	2.83	3.649 (2)	148

Symmetry codes: (i) 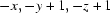; (ii) 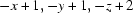. *Cg*1 is the centroid of the C1–C6 phenyl ring.

**Fig. 1.**
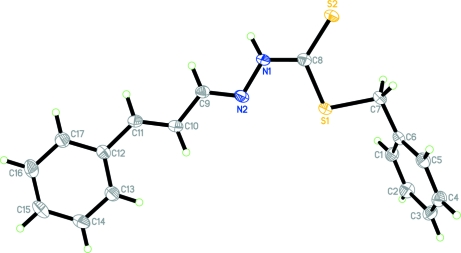
The asymmetric unit of the title compound, showing 50% probability displacement ellipsoids and the atomic numbering.

**Fig. 2.**
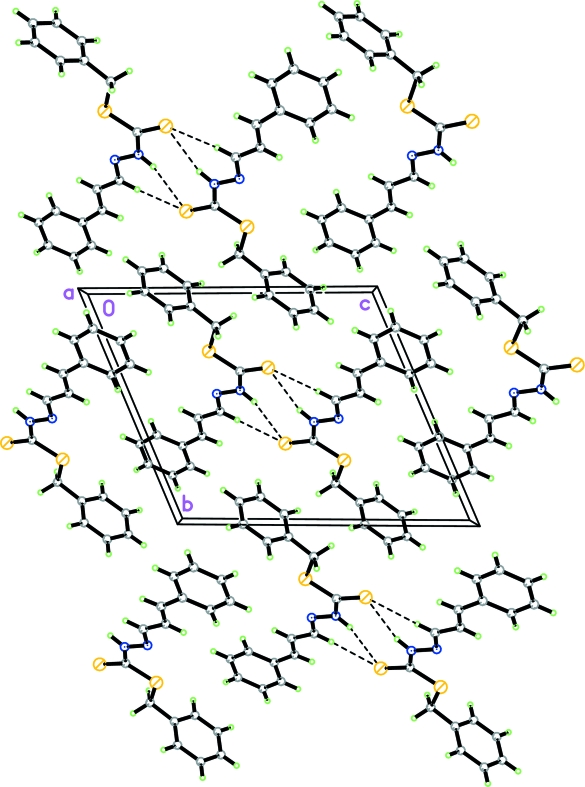
The crystal packing of the title compound, viewed along the a axis. Hydrogen bonds are shown as dashed lines.

## supplementary crystallographic information

### Comment

There has been immense interest in nitrogen-sulfur donor ligands since our report on *S*-benzyldithiocarbazate (SBDTC) (Ali & Tarafder, 1977). There have also been a number of reports of Schiff bases derived from SBDTC (Ali *et al.*, 2001, 2002, 2008; Chan *et al.*, 2008; Chew *et al.*, 2004; Tarafder *et al.*, 1978, 1981, 2001; Raj *et al.*, 2000). The intriguing coordination chemistry and increasingly important biomedical properties of ligands derived from SBDTC have also received much attention (Ali *et al.*, 2001, 2002; Crouse *et al.*, 2004; Tarafder *et al.*, 2001, 2008). The synthesis and structure of SBDTC have been reported previously (Ali & Tarafder (1977); Shanmuga Sundara Raj *et al.*, 2000). In continuation of our research, the title compound (I), a ligand with both N and S donor atoms, was synthesized and its crystal structure is reported here. (I) is likely to have biomedical properties similar to other nitrogen-sulfur donor ligands studied by our group.

In the title compound (Fig. 1), the 3-phenylprop-2-enylidene amide (N2/C9–C17) and benzyl groups (C1–C7) adopt *trans* and *cis* positions with respect to the terminal thione S2 atom about the C8-N1 and C8-S1 bonds, respectively. The 3-phenylprop-2-enylidene (C9–C17) and the dithiocarbazate (N1/N2/S1/S2/C8) fragments is essentially planar with maximum deviation 0.074 (2) Å for C11, while the dihedral angle between the 3-phenylprop-2-enylidene and the benzyl group is 77.78 (7)°. The bond lengths and angles are in normal ranges (Allen *et al.*, 1987). However the C═S distance of 1.7466 (17) Å is longer than the typical value of dithiocarbazate derivatives (Crouse *et al.*, 2004; Fun *et al.*, 2008; Shanmuga Sundara Raj *et al.*, 2000) but being intermediate between the values of 1.82 Å for a C—S single bond and 1.56 Å for a C═S double bond (Suton, 1965). The C9–N2 distance of 1.285 (2) Å indicates a double bond charactor. The bond angles S1–C8–S2 [124.67 (10)°] and N1–C8–S1 [113.76 (13)°] also agree with those observed in *trans-cis**S*-benzyl dithiocarbazate (Shanmuga Sundara Raj *et al.*, 2004).

In the crystal packing (Fig. 2), the molecules are linked by an N1—H1···S2^i^ hydrogen bond (symmetry code: i = -x, 1-y, 1-z) (Table 1) and a weak C9—H9A···S2^i^ interaction involving the terminal thione-S atom forming dimers that are arranged into sheets parallel to the *bc* plane. The crystal is also stabilized by C—H···π interactions (Table 1) involving the C1–C6 phenyl ring (centroid *Cg*1).

### Experimental

The title compound was synthesized by adding cinnamaldehyde (1.34 g, 10 mmol) to a solution of *S*-benzyldithiocarbazate (SBDTC) (1.98 g, 10 mmol) in absolute ethanol (60 ml) and the mixture was refluxed for 40 min. The yellow precipitate, which formed was separated and dried in vacuo over anhydrous CaCl_2_ (Yield: 2.1 g, 63%). Yellow needle shaped single crystals of (I) were obtained after recrystallization from absolute ethanol over 15 days; *M*.p 454 K.

### Refinement

The H1N1  hydrogen atom was located from a difference Fourier map and refined freely with isotropic displacement parameters. The remaining H atoms were positioned geometrically and allowed to ride on their parent atoms, with d(C—H) = 0.93 Å, for CH and aromatic, 0.97 Å, for CH_2_ and *U*_iso_ = 1.2*U*_eq_(C). The highest residual electron density peak is located at 0.96 Å from S1 and the deepest hole is located at 0.72 Å from S1.

### Crystal data

**Table:**

C_17_H_16_N_2_S_2_	*Z* = 2
*M**_r_* = 312.44	*F*_000_ = 328
Triclinic, *P*1	*D*_x_ = 1.320 Mg m^−^^3^
Hall symbol: -P 1	Melting point: 454 K
*a* = 5.4350 (3) Å	Mo *K*α radiation λ = 0.71073 Å
*b* = 11.6333 (7) Å	Cell parameters from 3570 reflections
*c* = 13.6289 (8) Å	θ = 1.9–27.5º
α = 66.869 (4)º	µ = 0.33 mm^−^^1^
β = 82.723 (4)º	*T* = 100.0 (1) K
γ = 87.520 (4)º	Needle, yellow
*V* = 786.04 (8) Å^3^	0.58 × 0.19 × 0.05 mm

### Data collection

**Table:**

Bruker SMART APEX2 CCD area-detector diffractometer	3570 independent reflections
Radiation source: fine-focus sealed tube	2870 reflections with *I* > 2σ(*I*)
Monochromator: graphite	*R*_int_ = 0.044
Detector resolution: 8.33 pixels mm^-1^	θ_max_ = 27.5º
*T* = 100.0(1) K	θ_min_ = 1.9º
ω scans	*h* = −7→7
Absorption correction: multi-scan(SADABS; Bruker, 2005)	*k* = −14→15
*T*_min_ = 0.829, *T*_max_ = 0.982	*l* = −17→17
16100 measured reflections	

### Refinement

**Table:**

Refinement on *F*^2^	Secondary atom site location: difference Fourier map
Least-squares matrix: full	Hydrogen site location: inferred from neighbouring sites
*R*[*F*^2^ > 2σ(*F*^2^)] = 0.035	H atoms treated by a mixture of independent and constrained refinement
*wR*(*F*^2^) = 0.093	*w* = 1/[σ^2^(*F*_o_^2^) + (0.0368*P*)^2^ + 0.3179*P*] where *P* = (*F*_o_^2^ + 2*F*_c_^2^)/3
*S* = 1.07	(Δ/σ)_max_ = 0.001
3570 reflections	Δρ_max_ = 0.29 e Å^−^^3^
194 parameters	Δρ_min_ = −0.27 e Å^−^^3^
Primary atom site location: structure-invariant direct methods	Extinction correction: none

### Special details

**Table:**

Experimental. The low-temperature data was collected with the Oxford Cyrosystem Cobra low-temperature attachment.
Geometry. All esds (except the esd in the dihedral angle between two l.s. planes) are estimated using the full covariance matrix. The cell esds are taken into account individually in the estimation of esds in distances, angles and torsion angles; correlations between esds in cell parameters are only used when they are defined by crystal symmetry. An approximate (isotropic) treatment of cell esds is used for estimating esds involving l.s. planes.
Refinement. Refinement of F^2^ against ALL reflections. The weighted R-factor wR and goodness of fit S are based on F^2^, conventional R-factors R are based on F, with F set to zero for negative F^2^. The threshold expression of F^2^ > 2sigma(F^2^) is used only for calculating R-factors(gt) etc. and is not relevant to the choice of reflections for refinement. R-factors based on F^2^ are statistically about twice as large as those based on F, and R- factors based on ALL data will be even larger.

### Fractional atomic coordinates and isotropic or equivalent isotropic displacement parameters (Å^2^)

**Table:**

	*x*	*y*	*z*	*U*_iso_*/*U*_eq_	
S1	−0.13630 (8)	0.74106 (4)	0.65560 (3)	0.02137 (13)	
S2	−0.23454 (8)	0.66804 (4)	0.47403 (4)	0.02329 (13)	
N1	0.1010 (3)	0.56987 (15)	0.60587 (12)	0.0209 (3)	
N2	0.2180 (3)	0.55500 (14)	0.69440 (11)	0.0212 (3)	
C1	−0.2508 (3)	1.02812 (17)	0.63242 (14)	0.0241 (4)	
H1A	−0.1133	1.0404	0.5812	0.029*	
C2	−0.2838 (3)	1.10363 (18)	0.68983 (15)	0.0270 (4)	
H2A	−0.1690	1.1664	0.6771	0.032*	
C3	−0.4872 (3)	1.08611 (18)	0.76620 (15)	0.0265 (4)	
H3A	−0.5087	1.1364	0.8054	0.032*	
C4	−0.6584 (3)	0.99380 (18)	0.78414 (15)	0.0261 (4)	
H4A	−0.7964	0.9824	0.8350	0.031*	
C5	−0.6253 (3)	0.91803 (17)	0.72668 (15)	0.0230 (4)	
H5A	−0.7413	0.8558	0.7393	0.028*	
C6	−0.4207 (3)	0.93393 (16)	0.65043 (14)	0.0203 (4)	
C7	−0.3781 (3)	0.84724 (17)	0.59218 (14)	0.0222 (4)	
H7A	−0.3250	0.8938	0.5164	0.027*	
H7B	−0.5287	0.8014	0.5993	0.027*	
C8	−0.0802 (3)	0.65301 (16)	0.57678 (14)	0.0199 (4)	
C9	0.3953 (3)	0.47537 (16)	0.71089 (14)	0.0205 (4)	
H9A	0.4371	0.4369	0.6628	0.025*	
C10	0.5303 (3)	0.44442 (16)	0.80163 (14)	0.0207 (4)	
H10A	0.4896	0.4829	0.8497	0.025*	
C11	0.7138 (3)	0.36108 (16)	0.81818 (14)	0.0209 (4)	
H11A	0.7506	0.3277	0.7662	0.025*	
C12	0.8636 (3)	0.31572 (16)	0.90742 (14)	0.0202 (4)	
C13	0.8492 (3)	0.36609 (17)	0.98578 (15)	0.0254 (4)	
H13A	0.7375	0.4298	0.9830	0.030*	
C14	0.9991 (4)	0.32206 (18)	1.06702 (15)	0.0285 (4)	
H14A	0.9889	0.3570	1.1182	0.034*	
C15	1.1645 (4)	0.22635 (19)	1.07320 (15)	0.0298 (4)	
H15A	1.2654	0.1972	1.1282	0.036*	
C16	1.1791 (3)	0.17447 (19)	0.99739 (15)	0.0306 (5)	
H16A	1.2890	0.1096	1.0016	0.037*	
C17	1.0301 (3)	0.21890 (18)	0.91488 (15)	0.0257 (4)	
H17A	1.0415	0.1836	0.8639	0.031*	
H1N1	0.129 (4)	0.519 (2)	0.5733 (18)	0.038 (6)*	

### Atomic displacement parameters (Å^2^)

**Table:**

	*U*^11^	*U*^22^	*U*^33^	*U*^12^	*U*^13^	*U*^23^
S1	0.0237 (2)	0.0250 (2)	0.0198 (2)	0.00424 (18)	−0.00880 (17)	−0.01190 (19)
S2	0.0242 (2)	0.0305 (3)	0.0194 (2)	0.00284 (19)	−0.00832 (17)	−0.0129 (2)
N1	0.0221 (8)	0.0265 (8)	0.0190 (8)	0.0024 (6)	−0.0071 (6)	−0.0128 (7)
N2	0.0218 (8)	0.0265 (8)	0.0165 (7)	−0.0001 (6)	−0.0052 (6)	−0.0090 (6)
C1	0.0193 (9)	0.0296 (10)	0.0230 (9)	0.0001 (8)	0.0002 (7)	−0.0106 (8)
C2	0.0238 (10)	0.0256 (10)	0.0316 (11)	−0.0046 (8)	−0.0010 (8)	−0.0114 (9)
C3	0.0255 (10)	0.0279 (10)	0.0311 (10)	0.0026 (8)	−0.0030 (8)	−0.0172 (9)
C4	0.0193 (9)	0.0321 (11)	0.0279 (10)	−0.0005 (8)	0.0007 (7)	−0.0136 (9)
C5	0.0182 (9)	0.0250 (10)	0.0260 (10)	−0.0033 (7)	−0.0041 (7)	−0.0094 (8)
C6	0.0189 (9)	0.0223 (9)	0.0195 (9)	0.0031 (7)	−0.0076 (7)	−0.0067 (7)
C7	0.0212 (9)	0.0258 (10)	0.0211 (9)	0.0027 (7)	−0.0081 (7)	−0.0095 (8)
C8	0.0197 (9)	0.0225 (9)	0.0174 (9)	−0.0025 (7)	−0.0018 (7)	−0.0076 (7)
C9	0.0199 (9)	0.0229 (9)	0.0212 (9)	−0.0018 (7)	−0.0027 (7)	−0.0109 (8)
C10	0.0230 (9)	0.0233 (9)	0.0185 (9)	−0.0023 (7)	−0.0039 (7)	−0.0102 (7)
C11	0.0226 (9)	0.0217 (9)	0.0207 (9)	−0.0029 (7)	−0.0034 (7)	−0.0103 (7)
C12	0.0179 (9)	0.0206 (9)	0.0209 (9)	−0.0033 (7)	−0.0032 (7)	−0.0063 (7)
C13	0.0279 (10)	0.0239 (10)	0.0245 (10)	0.0001 (8)	−0.0067 (8)	−0.0085 (8)
C14	0.0361 (11)	0.0285 (10)	0.0214 (10)	−0.0048 (8)	−0.0084 (8)	−0.0083 (8)
C15	0.0236 (10)	0.0373 (11)	0.0217 (10)	−0.0033 (8)	−0.0074 (8)	−0.0025 (9)
C16	0.0227 (10)	0.0351 (11)	0.0276 (10)	0.0061 (8)	−0.0033 (8)	−0.0058 (9)
C17	0.0243 (10)	0.0293 (10)	0.0229 (10)	0.0024 (8)	−0.0017 (7)	−0.0100 (8)

### Geometric parameters (Å, °)

**Table:**

S1—C8	1.7466 (17)	C7—H7A	0.9700
S1—C7	1.8187 (17)	C7—H7B	0.9700
S2—C8	1.6696 (18)	C9—C10	1.433 (2)
N1—C8	1.334 (2)	C9—H9A	0.9300
N1—N2	1.382 (2)	C10—C11	1.337 (2)
N1—H1N1	0.87 (2)	C10—H10A	0.9300
N2—C9	1.285 (2)	C11—C12	1.460 (2)
C1—C2	1.381 (3)	C11—H11A	0.9300
C1—C6	1.390 (3)	C12—C17	1.394 (2)
C1—H1A	0.9300	C12—C13	1.399 (3)
C2—C3	1.381 (3)	C13—C14	1.378 (3)
C2—H2A	0.9300	C13—H13A	0.9300
C3—C4	1.379 (3)	C14—C15	1.384 (3)
C3—H3A	0.9300	C14—H14A	0.9300
C4—C5	1.384 (3)	C15—C16	1.380 (3)
C4—H4A	0.9300	C15—H15A	0.9300
C5—C6	1.387 (2)	C16—C17	1.387 (3)
C5—H5A	0.9300	C16—H16A	0.9300
C6—C7	1.504 (2)	C17—H17A	0.9300
			
C8—S1—C7	102.56 (8)	N1—C8—S1	113.76 (13)
C8—N1—N2	120.49 (15)	S2—C8—S1	124.67 (10)
C8—N1—H1N1	118.0 (15)	N2—C9—C10	121.56 (16)
N2—N1—H1N1	120.9 (15)	N2—C9—H9A	119.2
C9—N2—N1	114.00 (14)	C10—C9—H9A	119.2
C2—C1—C6	120.70 (17)	C11—C10—C9	121.02 (16)
C2—C1—H1A	119.6	C11—C10—H10A	119.5
C6—C1—H1A	119.6	C9—C10—H10A	119.5
C1—C2—C3	120.11 (18)	C10—C11—C12	128.25 (16)
C1—C2—H2A	119.9	C10—C11—H11A	115.9
C3—C2—H2A	119.9	C12—C11—H11A	115.9
C4—C3—C2	119.76 (17)	C17—C12—C13	118.27 (16)
C4—C3—H3A	120.1	C17—C12—C11	118.96 (16)
C2—C3—H3A	120.1	C13—C12—C11	122.77 (16)
C3—C4—C5	120.16 (17)	C14—C13—C12	120.56 (17)
C3—C4—H4A	119.9	C14—C13—H13A	119.7
C5—C4—H4A	119.9	C12—C13—H13A	119.7
C4—C5—C6	120.65 (17)	C13—C14—C15	120.60 (18)
C4—C5—H5A	119.7	C13—C14—H14A	119.7
C6—C5—H5A	119.7	C15—C14—H14A	119.7
C5—C6—C1	118.61 (16)	C16—C15—C14	119.64 (17)
C5—C6—C7	120.57 (17)	C16—C15—H15A	120.2
C1—C6—C7	120.76 (16)	C14—C15—H15A	120.2
C6—C7—S1	105.49 (12)	C15—C16—C17	120.11 (18)
C6—C7—H7A	110.6	C15—C16—H16A	119.9
S1—C7—H7A	110.6	C17—C16—H16A	119.9
C6—C7—H7B	110.6	C16—C17—C12	120.81 (18)
S1—C7—H7B	110.6	C16—C17—H17A	119.6
H7A—C7—H7B	108.8	C12—C17—H17A	119.6
N1—C8—S2	121.57 (13)		
			
C8—N1—N2—C9	−177.45 (16)	C7—S1—C8—S2	−2.52 (14)
C6—C1—C2—C3	0.1 (3)	N1—N2—C9—C10	−177.41 (16)
C1—C2—C3—C4	−0.6 (3)	N2—C9—C10—C11	179.74 (17)
C2—C3—C4—C5	0.7 (3)	C9—C10—C11—C12	−177.74 (17)
C3—C4—C5—C6	−0.1 (3)	C10—C11—C12—C17	173.65 (18)
C4—C5—C6—C1	−0.5 (3)	C10—C11—C12—C13	−6.9 (3)
C4—C5—C6—C7	176.79 (16)	C17—C12—C13—C14	1.0 (3)
C2—C1—C6—C5	0.5 (3)	C11—C12—C13—C14	−178.40 (17)
C2—C1—C6—C7	−176.74 (17)	C12—C13—C14—C15	−0.7 (3)
C5—C6—C7—S1	−102.64 (16)	C13—C14—C15—C16	−0.1 (3)
C1—C6—C7—S1	74.54 (18)	C14—C15—C16—C17	0.6 (3)
C8—S1—C7—C6	−175.27 (12)	C15—C16—C17—C12	−0.2 (3)
N2—N1—C8—S2	−176.88 (13)	C13—C12—C17—C16	−0.6 (3)
N2—N1—C8—S1	3.0 (2)	C11—C12—C17—C16	178.89 (17)
C7—S1—C8—N1	177.58 (14)		

### Hydrogen-bond geometry (Å, °)

**Table:**

*D*—H···*A*	*D*—H	H···*A*	*D*···*A*	*D*—H···*A*
N1—H1N1···S2^i^	0.87 (2)	2.53 (2)	3.3714 (19)	165 (2)
C9—H9A···S2^i^	0.93	2.93	3.7264 (18)	144
C15—H15A···Cg1^ii^	0.93	2.83	3.649 (2)	148

Symmetry codes: (i) −*x*, −*y*+1, −*z*+1; (ii) −*x*+1, −*y*+1, −*z*+2.
